# An 11 kg Phyllodes tumor of the breast in combination with other multiple chronic diseases: Case report and review of the literature

**DOI:** 10.3892/ol.2013.1361

**Published:** 2013-05-22

**Authors:** ZHILONG ZHAO, JIA ZHANG, YUANYUAN CHEN, LANWAN SHEN, JIANSHENG WANG

**Affiliations:** 1 Department of Surgical Oncology, First Affiliated Hospital, Xi’an Jiaotong University, Xi’an, Shaanxi 710061;; 2 Department of Surgical Oncology, Bao Ji Central Hospital, Bao Ji, Shaanxi 721000, P.R. China

**Keywords:** breast, phyllodes tumor, chronic diseases, cancer

## Abstract

Phyllodes tumors (PTs) of the breast are a rare type of tumor that account for <1% of breast tumors in females and usually present as a large lump of 3–5 cm in size. Surgery is the first line of treatment for PTs, and chemotherapy and irradiation may be useful in certain patients but not all. In the present study, the case of a 63-year-old female patient with a huge PT in the left breast is described. On physical examination, the patient presented with a huge mass of ∼45 cm in diameter, weighing ∼11 kg, and a composite of multiple chronic diseases. The breast and pectoris major and minor were excised. Post-operatively, the patient recovered well and to date there has been no evidence of local recurrence or distant metastasis.

## Introduction

Phyllodes tumors (PTs) of the breast are rare biphasic fibroepithelial tumors, characterized by the proliferation of stromal and epithelial cells (1,2). They account for 0.4–1% of all breast tumors and occur most frequently between the ages of 40 and 50 years (3). In the majority of cases, the tumor grows faster than any other tumor would; PT usually presents as a large lump of 3–5 cm in size when patients visit a doctor. The current study presents the case of a patient with an ∼11 kg PT measuring 45 cm at the maximum diameter, who exhibited a composite of multiple chronic diseases. The study also presents a review of the literature. We propose that this report may be useful for the diagnosis and treatment of PT. Written informed consent was obtained from the patient.

## Case report

**Figure 1.**
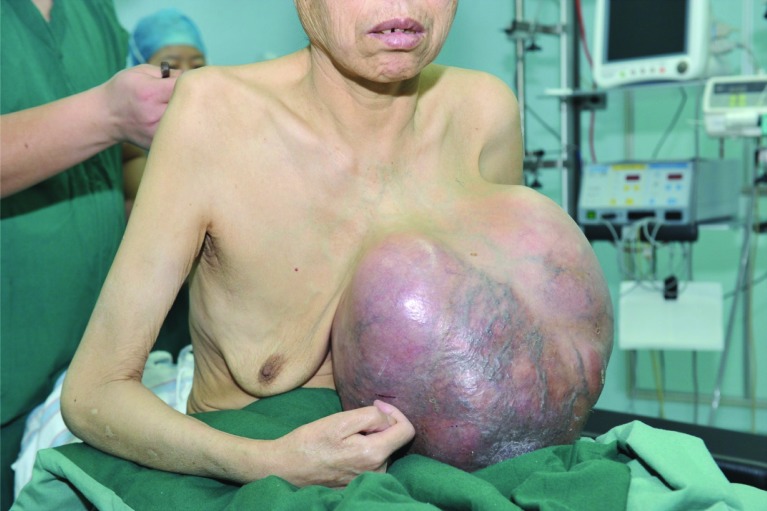
Patient presented with a massive tumor of the left breast prior to surgery.

A 63-year-old female patient was admitted to the Galactophore Department, Bao Ji Central Hospital with a huge lump in the left breast. The patient stated that they had identified a thumb-sized lump two years earlier, but that they had ignored it. From July 2011, the patient observed that the lump was growing rapidly; in February 2012 the lump had become so large that the patient’s left chest was entirely covered with a solid, irregularly-shaped, somewhat moveable mass of tremendous size and the patient was barely able to lie in the prone position. Family members then finally convinced the patient to seek medical attention. Upon physical examination, the patient presented with an elastic hard mass in the left breast that was 45 cm in diameter, with a circumference of 97 cm (Fig. 1). Rapid growth had resulted in shiny stretched skin that was translucent, showing underlying extremely enlarged veins with sections of the circuitous veins about to undergo ulceration, although no bleeding or discharge was noted. The lymph nodes in the axillary and other superficial areas were not palpable. The patient’s previous medical history revealed that they had high blood pressure, diabetes and atrial fibrillation. Moreover, the patient had suffered from a cerebral infarction; the patient’s right side was paralyzed and communicative disorders had appeared two years previously. The patient was unable to care for herself due to the cerebral infarction. The patient’s medical history was negative for allergies, smoking, drinking, contagions and serious addiction. Furthermore, there was no family history of breast cancer on either side of the patient’s family.

**Figure 2.**
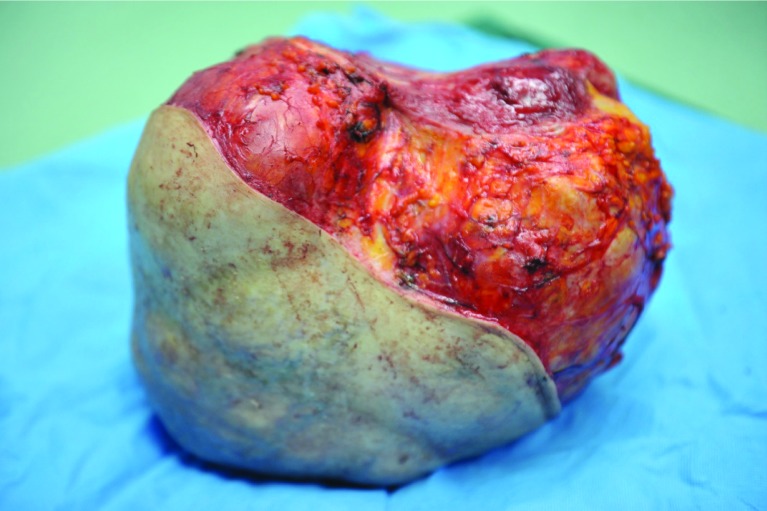
Resected Phyllodes tumor (PT) weighing 11 kg and measuring 36×40×18 cm.

**Figure 3.**
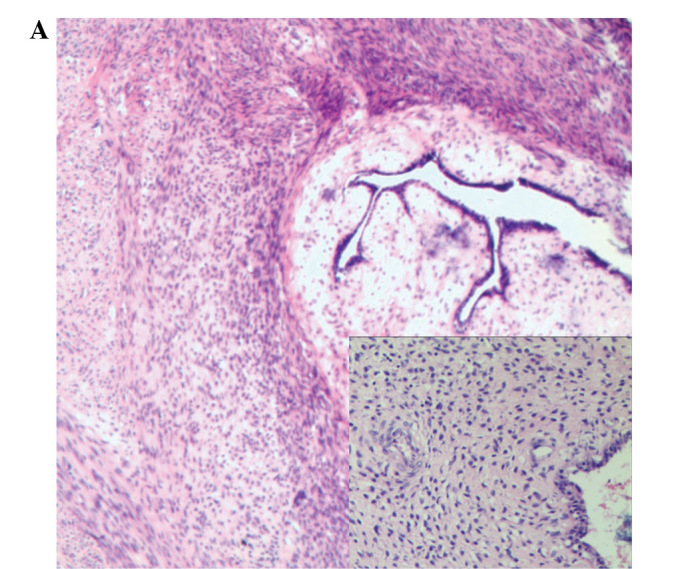
Hematoxylin and eosin staining of the breast tissue revealing an epithelial component with a leaf-like pattern, but no cellular atypia. Magnification, ×100 (inset, ×400)..

Due to the non-compressible nature of the mass and the size of the tumor, regular breast examinations, including mammography and ultrasound studies, could not be used, while a computed tomography (CT) scan of the chest revealed a solid mass. Total body scintigraphy was negative. Comprehensive examinations and detailed assessments were additionally performed on the patient due to the presence of high blood pressure, diabetes, atrial fibrillation and cerebral infarction. In order to ensure the long-term success of the treatment, a consultation with the Respiratory Medicine, Vasculocardiology, Neurology and Anesthesiology departments was organized. Antihypertensive, glycemic control, oxygen uptake and antiarrhythmic therapies were administered prior to surgery. The pectoris major and minor had atrophied and were tightly adhered to the tumor due to long-term compression, so the surgeon decided to resect the mass along with these muscles. The mass was resected subsequent to 3 hours of surgery (Fig. 2). The post-operative pathology report showed an extremely large grey mass, weighing 11 kg and measuring 36×40×18 cm, which was covered by ∼32×35 cm of skin tissue. When the tumor was cut into halves along the nipple, it was observed that the majority of the tumor tissue was a myxoid, lobulated mass with multiple hollow cavities, thick, yellow, viscous fluid and certain necrotic areas. The microscopy findings showed extensive necrosis and the majority of the tumor tissue exhibited an epithelial component with a leaf-like pattern, but no cellular atypia. The final histopathological diagnosis was PT of the benign type (Fig. 3).

In view of the benign nature of this tumor, no chemotherapy or radiotherapy was administered following surgery. The patient recovered well and to date there has been no evidence of local recurrence or distant metastasis.

## Discussion

PTs, also known as cystosarcoma phyllodes, were first named by Johannes Muller (4,5). Muller described cystosarcoma phyllodes as leaf-like masses with a cyst-like shape and complete envelopes. There were however, numerous other names used to label this type of case. In 1982, the World Health Organization (WHO) decided to use the term PT to describe these cases in the official WHO’s standard classification of breast diseases (6), and this term is now commonly used.

PTs are rare tumors of the breast that account for 0.4–1% of all breast tumors, and the average age of the affected patients is 45 (3). At present, the exact causes of PTs remain unknown, although the majority of researchers consider that PTs are likely to have similar epidemic factors to fibroadenoma of the breast, which is associated with estrogen secretion and metabolic disorders. With regard to the clinical and histological aspects, small PTs have apparent similarity to fibroadenomas (7).

In 1982, PTs were classified by the WHO as benign, borderline and malignant, on the basis of cell density, atypia, mitotic figures, tumor borders and hemorrhagic necrosis. Malignant tumors (either low- or high-grade) are common in elderly females and exhibit frequent metastasis (lymphatic and blood-borne) with a high mortality rate (8). Malignant PTs have a high rate of recurrence and the axillary and supraclavicular lymph nodes are commonly enlarged (9). However, 70–90% of all PTs are benign tumors that do not metastasize, although they exhibit continuous growth and rapidly increase in size in a short time. Due to the overlapping histological and clinical features between small PTs and fibroadenomas, there are no known tumor markers or blood tests to aid in the confirmation of the diagnosis. Consequently, it is easy to misdiagnose the majority of PTs as fibroadenomas. The differentiation of PTs from benign fibroadenomas is difficult using ultrasound, mammograms and magnetic resonance imaging (MRI). Adamietz *et al* (10) suggested that real-time elastography may be sufficient to differentiate between these two lesions. The authors proposed that all PTs have a similar elastic pattern with an elastic center and inelastic outer limits, although this pattern was also observed in 5% of all fibroadenomas. A study by Bandyopadhyay *et al* (11) attempted to make the distinction from a cytologists’ perspective. The study suggested that the size, cellularity of stromal fragments and proportion of spindle cells in the background are significant features in such differentiation. Spindle cells appear to be present in large numbers in PTs compared with fibroadenomas.

The primary treatment for PTs is surgery, although the most suitable type of surgery is debatable (wide local excision or total mastectomy). In certain cases, there may be a requirement to resect the pectoris major and minor. If the mass is >5 cm or malignant, wide local excision is recommended. Although chemotherapy, radiotherapy and local lymph node dissection are not used in conventional treatment modalities for PTs, they may be useful for patients with aggressive tumors, positive margins and high mitotic rates, as well as for those with recurrence (12). The recurrence rate in benign tumors (5–15%) is lower than in malignant tumors (20–30%). Certain studies have shown that the overall five-year disease-free survival rate ranges from 78–91% and that the resection margin and size of the tumor may be key factors (13).

In summary, PTs of the breast are rare and the present malignant PT is notable due to its large size and weight of ∼36×40×18 cm and 11 kg, respectively, in combination with the presence of other chronic diseases. We suggest that this report may be useful for the diagnosis and treatment of future PT patients.
